# Long-term body tactile stimulation reduces aggression and improves productive performance in Nile tilapia groups

**DOI:** 10.1038/s41598-022-24696-3

**Published:** 2022-11-24

**Authors:** Ana Carolina dos Santos Gauy, Marcela Cesar Bolognesi, Eliane Gonçalves-de-Freitas

**Affiliations:** 1 Departamento de Ciências Biológicas, Instituto de Biociências, Letras e Ciências Exatas, Universidade Estadual Paulista (UNESP), Rua Cristóvão Colombo, 2265, São José do Rio Preto, SP 15054-000 Brazil; 2 CAUNESP-Centro de Aquicultura da UNESP, Jaboticabal, SP Brazil

**Keywords:** Zoology, Animal behaviour

## Abstract

One concern of the Anthropocene is the effects of human activities on animal welfare, revealing the urgency to mitigate impacts of rearing environments. Body tactile stimulation (TS), like massage therapy, has emerged as an enrichment method to counteract stress and anxiety in vertebrates. In the current study, we evaluated the effects of long-term TS on four-member groups of male Nile tilapia, a worldwide reared species whose socially aggressive behavior is an essential source of stress. We placed a rectangular PVC frame fitted with vertical plastic sticks sided with silicone bristles in the center of aquarium to enable the fish to receive body TS when passing through the bristles. A similar apparatus without bristles was used as the control. Fish subjected to TS for 21 days showed a gradual lowering of overt fights over time, but with no reduction in cortisol or androgen levels. Nevertheless, TS improved the specific growth rate, maintained balanced length/weight gain, and increased feed efficiency, probably owing to the lowered energy expenditure during fights. Thus, we show for the first time that long-term TS provided by a simple device can be used as a tool to improve the welfare and productive performance of territorial fish.

## Introduction

One important concern of the Anthropocene is the effect of human activities on animal welfare, as they are reared in artificial environments for different purposes, from scientific research to livestock farming. The growth of global aquaculture has necessitated that due importance be given to fish welfare, which is now viewed as important as mammalian welfare^1^. Attention to the life quality of fish has increased, particularly in this century, with studies showing that fish are sentient (i.e., capable of feeling pain and fear) and show complex behavior, such as social interaction and cooperation, as well as complex cognitive abilities^2,3^.

Besides husbandry for food, fish are reared for scientific research, recreation, and ornamental trading. Regardless of the purpose, stress in the rearing environment, such as confinement, netting, grading, slaughtering, transportation, and non-natural aggressive interactions in territorial species, can impair fish welfare^4^. These stressors are perceived by the central nervous system which activates the sympathetic branch of the autonomic nervous system that triggers a fight-and-flight response, stimulating rapid cardiac and respiratory adjustments^5^. This is followed by a surge in adrenaline secretion from chromaffin tissues (specifically, the adrenal medullae in mammals), leading to decomposition of glycogen into glucose for quick usage by active tissues, among other effects. Therefore, the hypothalamus-pituitary-interrenal (HPI) axis increases the synthesis and secretion of glucocorticoid hormones, mainly cortisol, triggering metabolic changes, allowing the fish to cope with the stressors^5^. Although stress is an adaptive response of animals facing environmental challenges, the intensity and persistence of some stressors can lead to distress, characterized by elevated cortisol levels that impair growth and reproduction^6^ and cause neuron death and depression of the immune system^7,8^. This calls for development of solutions to reduce stress and distress in artificial environments to improve the quality of life of fish.

Stress alone does not determine the welfare, despite being a key factor. According to Huntingford et al.^9^, there are several other indicators of health and basic functioning of the organism, such as feeding, growth, and reproduction (function-based approach); absence of fear, pain, and suffering (feelings-based approach); and expression of the natural behavior repertoire (nature-based approach). In this context, a set of indicators are ideally more informative about the status of fish welfare. While working with one approach, other approaches can also be inculcated, particularly by offering conditions that bring positive valence to the individuals. Indeed, positive states must better represent welfare states in animals^10,11^.

Recent studies have shown that body tactile stimulation (TS), which is analogous to a massage therapy in humans, is a way to provide positive states in non-human vertebrates. TS is a type of natural or artificial mechanical stimulus applied to parts of an animal body that positively affects the recipient and acts as a sensory enrichment^12^. In adult humans, TS is known to decrease stress, improve immune responses, and elevate serotonin levels^13^. It also lowers stress in non-human mammals such as beef cattle^14^, piglets^15^, lambs^16^, dairy cows^17^, and dogs^18^. TS prevents distress and depressive states in neonatal rats^19^ and counteracts the negative effects of isolation by increasing body weight, social interaction, and decreasing anxiety in isolated voles^20^. Silva et al.^21^ reported reduced fear and soothing effect that lasted long in calves after long-term TS by brushing. Overall, these studies showed that TS can improve the welfare in mammals through several mechanisms.

TS has also been associated with positive effects in fish. During intraspecific cooperation between reef fishes, one organism (cleaner) can naturally provide TS to another (client) while removing parasites and maintaining cooperation^22^. TS is also part of the mechanism that maintains social interaction in both larval and adult forms of *Corydoras aeneus*^23^. Besides natural function, Soares et al.^24^ showed that TS from an artificial model of cleaner fish decreased stress in the client surgeonfish, *Ctenochaetus striatus.* Furthermore, artificial TS has been shown to minimize fear in zebrafish^25^ and reduce aggression in isolated male Nile tilapia^26^.

TS is a type of mechanosensory activity that is processed via sensory cells to the spinal cord and is interpreted in the encephalic regions of the central nervous system. In fish, the mechanosensory cells are distributed along the lateral line, fins, and other regions of the skin^27,28^. Similar to mammals, mechano-stimulation seems to be associated with serotonergic pathways^29^. Because the neural pathways of mechanical sensation and their connection with the hypothalamus-pituitary axis are well conserved in vertebrate animals, it is plausible to assume that the effects of TS found in mammals would also be found in teleost fishes.

**Figure 1:**
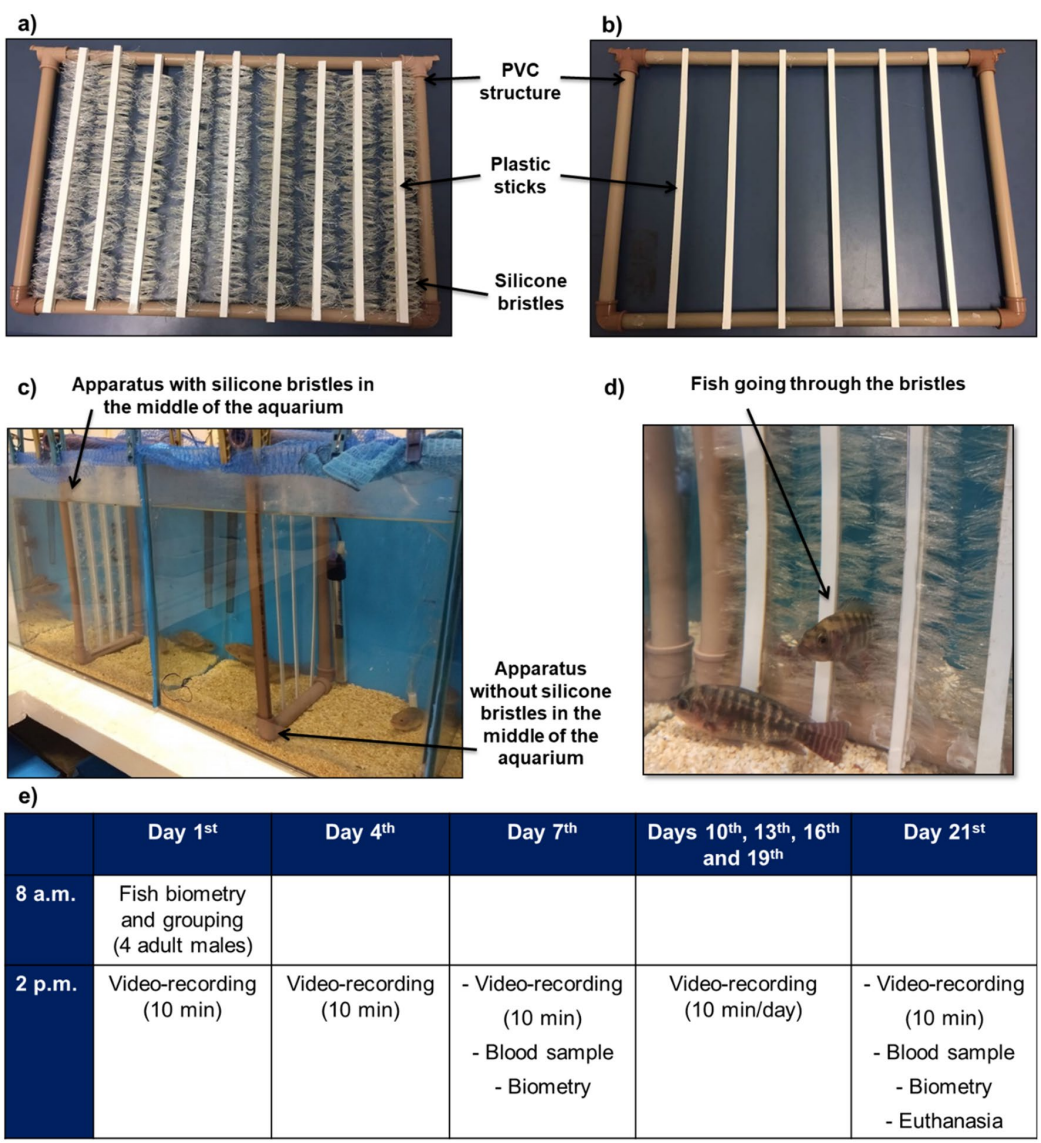
Tactile stimulation apparatus and protocol timeline. The apparatus was a rectangular PVC frame fitted with plastic sticks with silicone bristles in their sides to promote tactile stimulation (**a**) and without silicone bristles as the control (**b**). The apparatus was introduced into the middle of each experimental aquarium as per treatment (on the left, aquarium of the TS treatment with silicone bristles; on the right, aquarium of the control treatment without silicone bristles) (**c**). An example of fish going through the silicone bristles (**d**) is shown. The animals were first measured and weighed, then marked with colored elastomer, and assigned in four-male groups to one of the treatments, whose aquaria was already prepared with the respective apparatus. Three walls of each experimental aquarium were covered with opaque blue plastic to avoid visual contact with neighboring areas; only the front part was open for observations and filming. Groups were video-recorded every 3 days in the afternoon, and blood was sampled on days 7 and 21 for cortisol and androgen assay. The protocol timeline (**e**) is summarized accordingly.

Most TS studies in fish have been carried out using isolated animals over short periods of 10 days or less^24,26^. Undeniably, the efficiency of TS in improving fish welfare under artificial rearing should be tested over longer durations and in larger groups, as social aggressiveness is an important source of stress and energy expenditure in rank-based social fish such as Nile tilapia. It is the third common species worldwide to be reared in aquaculture and is widely used as a model for testing behavioral and physiological hypotheses. Even though aggressive interactions are part of their natural behavior, some common practices in aquaculture can increase their aggressiveness to a non-natural level. This, in turn, can increase social stress to a non-adaptive state, impairing the basic functioning of these fish^30^. In addition, it can impact growth and feed efficiency, decreasing the productive performance of animals. Therefore, this study aimed to test whether long-term TS has a positive effect on the welfare of Nile tilapia social groups by reducing aggression and social stress levels and improving productive performance. For this purpose, we analyzed groups of four male fish for 21 days under TS, using an apparatus previously developed for this goal^26^ (Fig. 1). We predicted that fish under TS will show reduced aggressive interaction in the group, decreased cortisol levels, increased growth rates (SGR), and better feed efficiency rate (FER). Furthermore, we expected to find reduced levels of androgens, which are associated with aggressive behavior in fish and tend to be higher in groups experiencing increased aggression and social instability^31^, namely testosterone (T) and 11- ketotestosterone (11-KT).

## Results

### Number of crossings by the tactile and non-tactile apparatus

**Figure 2:**
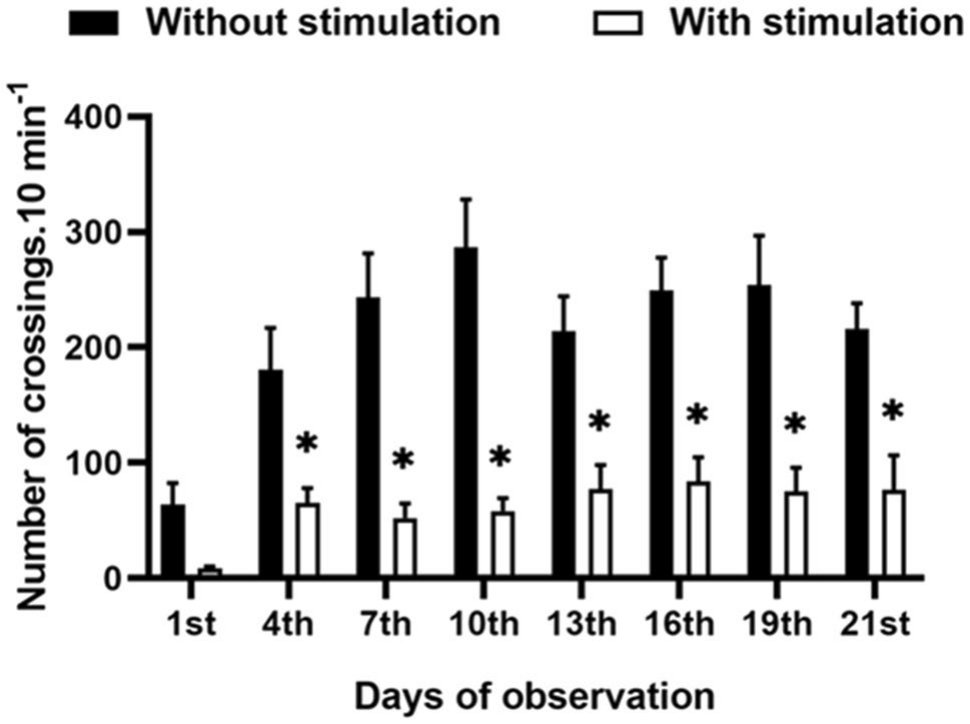
Number of crossings by the tactile and non-tactile apparatuses. The sum of the four individuals’ crossings by the apparatus at each observation day. Asterisks indicate significant differences between treatments after Mixed model ANOVA from day 4 to 21. The first day was not computed in the analysis because it did not reach the parametrical assumptions after data transformation. Data are mean ± S.E.

Fish crossed the apparatus more times in the absence of the tactile bristle than in those subjected to TS (F_(1,15)_ = 40.26, *p* = 0.000013), with no differences within treatments over time (F_(1,6)_ = 6.42, *p* = 0.24) (Fig. 2).

### Aggressive interaction

**Figure 3:**
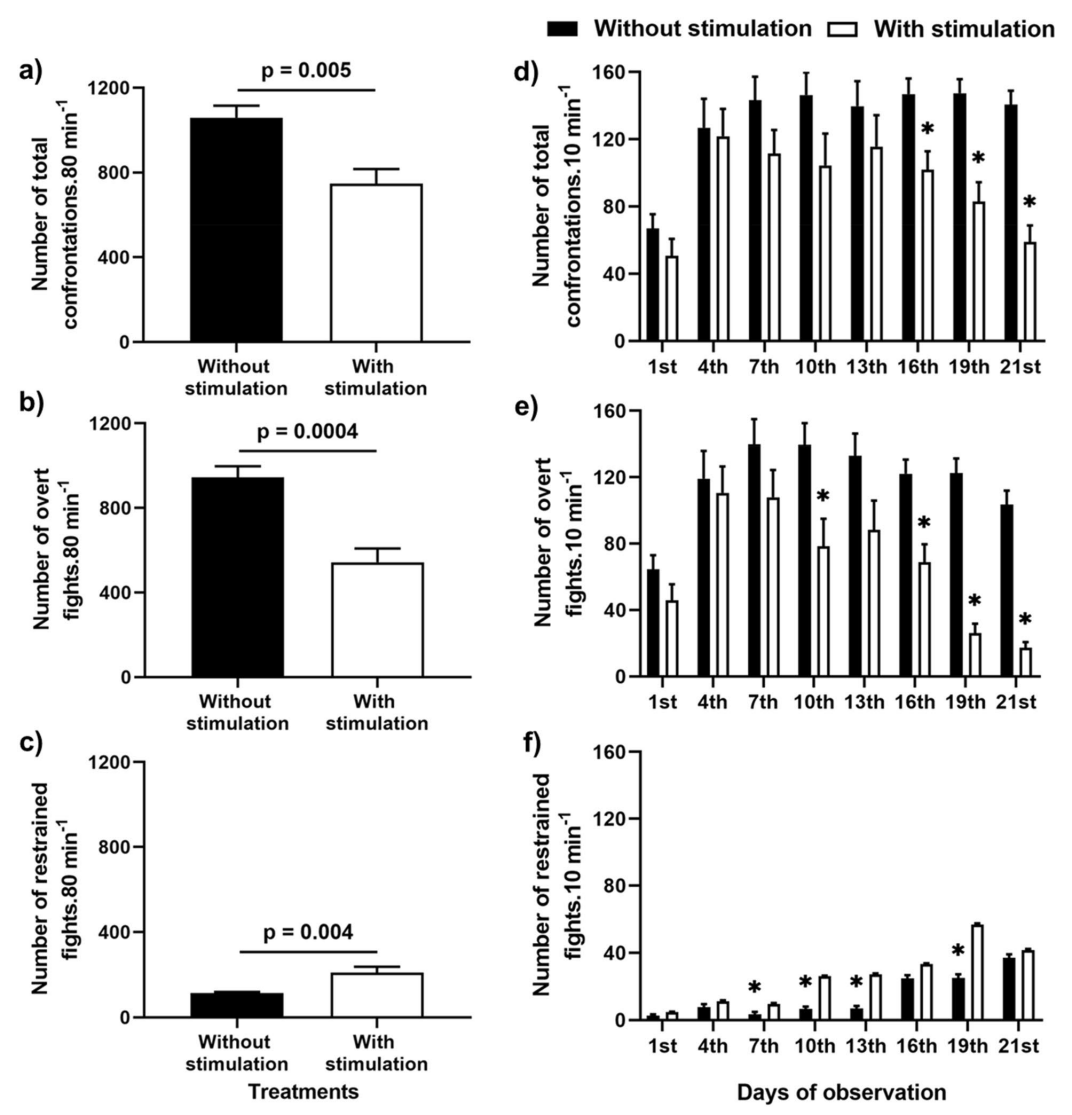
Number of fights between treatments and over the time. Sum of 21-days total confrontations (overt + restrained fights) (**a**), overt (**b**), and restrained fights (**c**). Number of total (**d**), overt (**e**) and restrained fights (**f**) over 21 days. The p values over the bars were results after Mixed Model ANOVA. Asterisks mean significant differences between treatments after contrasting by planned comparisons. Data are mean ± S.E.

Overall, aggressive interactions were reduced by TS. We found significant statistical interaction (between-within treatments) in the number of overt fights (F_(7,105)_ = 3.89, *p* = 0.0008), restrained fights (F_(7,105)_ = 4.00, *p* = 0.0006), and total confrontations (overt fights + restrained fights) (F_(7,105)_ = 2.17, *p* = 0.04) between the two treatment groups. The number of total confrontations and overt fights was higher in the treatment groups without TS (F_(1,15)_ = 10.54, *p* = 0.005; Fig. 3a, and F_(1,15)_ = 20.67, *p* = 0.0004; Fig. 3b), whereas the number of restrained fights was lower in these groups (F_(1,15)_ = 11,85, *p* = 0.004; Fig. 3c). Contrasts by planned comparisons showed significant differences over time between treatments from 16^th^ day for total confrontations (Table 1; Fig. 3d). However, on comparing overt and restrained fights, differences were found earlier, from 10 and 7^th^ day, respectively (Table 1; Figs. 3e,f).

**Table 1:** F and p values from planned comparisons applied to the number of aggressive interactions on the treatments with *vs.* without tactile stimulation over time.

Days of observation	Aggressive behavior					
	Overt fights		Restrained fights		Total confrontations	
	F_(1,15)_	*p*	F_(1,15)_	*p*	F_(1,15)_	*p*
1st	1.86	0.19	2.79	0.11	1.34	0.26
4th	0.13	0.72	3.39	0.08	0.04	0.84
7th	1.86	0.19	10.51	**0.005**	2.40	0.14
10th	7.30	**0.02**	8.91	**0.009**	2.70	0.12
13th	3.43	0.08	11.99	**0.003**	0.88	0.36
16th	12.46	**0.003**	1.38	0.26	8.60	**0.01**
19th	93.25	** < 0.0001**	12.93	**0.003**	17.37	**0.0008**
21st	116.40	** < 0.0001**	0.06	0.80	35.74	**0.00003**

Significant difference between TS and control are bold.

### Hormones

**Figure 4:**
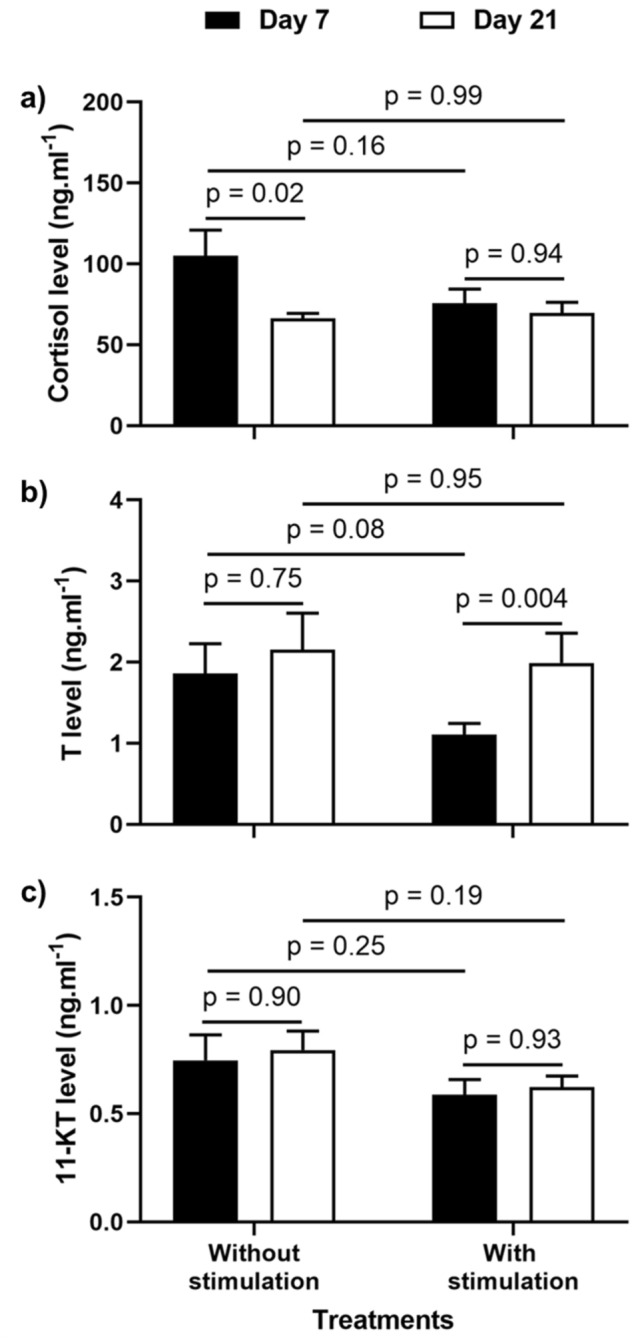
Steroid hormones levels between treatments on days 7 and 21. Plasma Cortisol (**a**), Testosterone—T (**b**), and 11-ketotestosterone—11-KT (**c**) levels. The p values over the bars are from contrasts by planned comparisons. Data are mean ± S.E.

We analyzed the differences in the mean levels of cortisol, T, and 11-KT per group of four fish between treatments with and without TS, as well as between the 7^th^ and 21^st^ day. Cortisol levels were similar between the treatments (F_(1,15)_ = 1,96; *p* = 0.18), but a difference was observed within treatments (F_(1,15 )_ = 5,39; *p* = 0.03). Contrasts by planned comparisons showed a significant reduction on 21^st^ day only in the control group (Fig. 4a). T levels were similar between treatments (F_(1,15)_ = 1,64; *p* = 0.22) and across days of observation (F_(1,15)_ = 3.47; *p* = 0.08), although planned comparisons showed a significant increase from 7^th^ to 21^st^ day in TS-treated groups (Fig. 4b). No significant differences were found for 11-KT levels, either between (F_(1,15)_ = 3.12; *p* = 0.10) or within (F_(1,15)_ = 0.43; *p* = 0.52) treatments, even after planned comparison analysis (Fig. 4c).

### Productive performance

**Figure 5:**
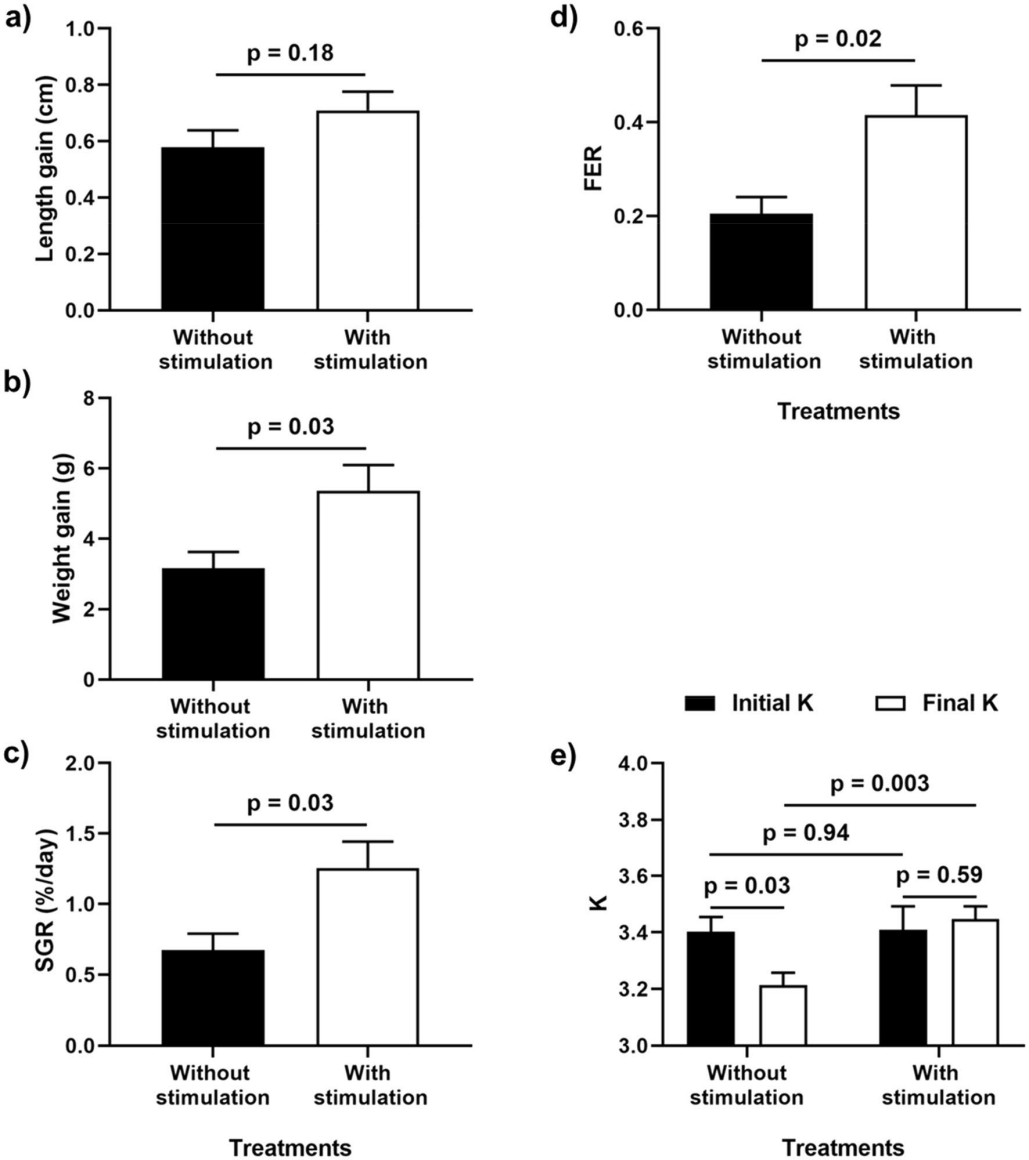
Productive performance. Length gain (a), weight gain (**b**), specific growth rate—SGR (**c**), and feed efficiency rate—FER (**d**) after independent t test. Condition factor—K (**e**) in the 1^st^ and 21^st^ days, after contrasting by planned comparisons. Data are mean ± S.E.

Fish weight and standard length (SL) increased significantly from 1 to 21^st^ day in both treated and control groups, although no significant differences were observed between treatments for these variables (weight statistical interaction: treatments *vs*. days: F_(1,15)_ = 5.25, *p* = 0.04; SL within treatment: F_(1,15)_ = 5.25, *p* < 0.00001; SL between treatment F_(1,15)_ = 5.25, *p* = 0.18). See Table 2 for the post-hoc results. Despite length gain being similar between TS-treated and control groups (t_(15)_ =  − 1.39, *p* = 0.18; Fig. 5a), fish subjected to TS showed higher weight gain, SGR and FER(t_(15)_ =  − 2.30, *p* = 0.03; t_(15)_ =  − 2.38, *p* = 0.03; t_(15)_ =  − 2.55, *p* = 0.02, respectively; Fig. 5b–d). Furthermore, a marginally significant interaction between *vs.* within treatments was found for condition factor (K) (F_(1,15)_ = 4.40, *p* = 0.053). Planned comparisons (Fig. 5e) showed no difference in the initial K between treatments (F_(1,15)_ = 0.005, *p* = 0.94), but the final one was higher for TS (F_(1,15)_ = 12.54, *p* = 0.003). It decreased from the beginning in the control group (F_(1,15)_ = 5.22, p = 0.03) and remained the same in the TS group (F_(1,15)_ = 0.29, *p* = 0.59). The proportion of group survival (without any death among the four members) was higher in the TS group (77%) than in the control group (50%) (Goodman's binomial, *p* < 0.05).

**Table 2:** Post hoc comparisons between treatments with and without TS, and within treatments (day 1 and day 21) for the Weight and Standard Length. Data are mean ± SE.

Treatment	Standard Length (cm)			Body Weight (g)		
	Initial	Final	*p* within treatments	Initial	Final	*p* within treatments
Without TS	8.59 ± 0.17	9.17 ± 0.12	**0.0002**	21.67 ± 1.17	24.83 ± 0.89	**0.003**
With TS	8.10 ± 0.22	8.81 ± 0.25	**0.0002**	18.51 ± 1.62	23.87 ± 1.69	**0.0002**
*p* between treatments	0.48	0.71		0.55	0.98	

P values after Tukey post hoc test for unequal N. Significant differences are bold.

## Discussion

In this study, we showed that artificial TS decreased social aggression in groups of cichlid fish. Although cortisol levels did not change, TS improved fish survival, growth rate, and feed efficiency, and maintained uniform growth; all of which can be attributed to reduced fights. Thus, artificial TS was shown for the first time to improve fish welfare and productive performance.

TS-treated fish crossed the respective TS apparatus less than the control fish, which could be seen as an aversive reaction to such a device. However, while testing the efficiency of a similar apparatus, Gauy et al.^12^ had shown that Nile tilapia males spontaneously chose to pass through the bristles despite having the option to avoid them. They also demonstrated their motivation to achieve TS. Nonetheless, the frequency of choice for an area without bristles is higher^12^. Thus, the silicone bristles may have caused some resistance, making it easier for the fish to pass through open control areas. Nevertheless, this did not prevent the effect of TS on reducing aggressiveness and improving productive performance of fish here.

The TS apparatus in the middle of the aquarium clearly reduced aggressiveness from the 10^th^ day after grouping, which can be interpreted in two ways: either as a positive effect of body TS (as predicted) or as a visual barrier, reducing the probability of individual encounters. The data do not fully support the second possibility because, although visual signals are pivotal in the social communication of Nile tilapia^32^, the frequency of aggressive interaction was similar between TS and control groups during the first week of grouping. Furthermore, chemical communication, another important signal for social rank recognition in Nile tilapia^33^ and other cichlids^34^, was not prevented in this experimental design. We also observed an increase in restrained fights marked by visual displays. Therefore, it is unlikely that reduced aggressiveness can only be explained by changes in visual perception. Bolognesi et al.^26^ observed that isolated Nile tilapia males that received the same type of TS were less aggressive than individuals that did not experience TS when faced with another fish in a neutral arena with no tactile apparatus. We could also suppose that the TS apparatus better divided the aquarium into two territories. If so, we would expect a reduction in the number of crossings through the apparatus. However, the number of crossings was constant, meaning that individuals approached each other constantly, whereas overt fights were reduced. Nonetheless, this explains the enhancement in restraining fights. To sum up, these results indicate that the effects on social aggression observed are consequences of TS. In social rank-based fish, overt fights are usually reduced and replaced by restrained ones^35^, thus animals signal their social rank without intense fights and resultant energy expenditure or injury^36^. Therefore, we suggest that there is a cumulative effect of TS on aggressiveness and social stability in Nile tilapia.

No information is available in the literature regarding the physiological effects of body TS on fish aggressiveness. Some changes in brain monoamines, such as dopamine, oxytocin, and serotonin, have been observed after massage therapy in humans^13^ and non-human mammals^37^. Previous studies on the involvement of serotoninergic pathways revealed that serotonin blockade reduces aggression towards conspecifics in *Labroides dimidiatus*^38^ and client fish^39^, indicating that these mutualistic interactions in labroid fish are examples of aggressive control by tactile interaction and, to some extent, by the serotonin system. In non-mutualistic species, serotonin also decreases aggressive interactions^40–42^. Nile tilapia males that were fed a diet enriched with tryptophan (a serotonin precursor) also showed decreased aggression^43^. Further, as serotonin pathways in fish and mammals are similar^29,44^, this system could be associated with the reduced aggression observed during TS in our study. This mechanism, however, still needs to be unveiled.

TS alone is an efficient stress reducer in mammals^13,19^ and fish^24^. Hence, we expected to observe this effect, as reduced cortisol is also associated with decreased aggressiveness in fish^45^. However, our results showed that TS did not reduce cortisol levels of Nile tilapia. Similar results were obtained in a study on feedlot steers by Park et al.^37^, where TS reduced aggressive interaction without lowering the cortisol levels. Thus, separate mechanisms may be involved during TS and stress regulation. However, although cortisol is a standard stress indicator in fish and mammals, complementary indicators of metabolic activity, such as blood glucose and liver glycogen concentration, should be used to better address stress levels^46^. Nevertheless, the difference in cortisol levels observed between the control and TS on day 7 may be related to the animals' adjustment to the social environment, caused by faster social stability in TS . Furthermore, we found a negative correlation between cortisol levels and the number of crossings through the apparatus only in TS treatment. Hence, we suggest that TS, to some extent, is associated with a rapid decrease in social stress in Nile tilapia.

The fights, maintenance of dominance hierarchy, and social stress produce considerable energy costs in Nile tilapia, which can impair other essential activities such as growth^47^. Cortisol is usually involved in the mechanism of reducing growth by aiding breakdown of reserve substrate and protein degradation when an individual reaches the distress phase^48^. In this study, we found no negative SGR, both in TS-treated and control groups showing that, although we found no effects of TS on cortisol levels, the animals did not enter the distress phase.

In addition to cortisol, androgens have been identified as regulators of social aggression in cichlid fish^49,50^; therefore, we expected a reduction in androgen levels following reduced fights in the group. However, these changes depend on species, social rank, and social stability^49,51^. Dominant Mozambique tilapia males (*Oreochromis mossambicus*), for instance, show higher levels of T and 11-KT than subordinates^49^ after fight resolution, but intense aggression is not linked with 11-KT levels^52^. Groupwise, no difference was observed in the level of 11-KT between and within the groups, but T was increased in fish subjected to TS, suggesting that the reduced metabolism of T to 11-KT is associated with reduced aggression in Nile tilapia. Additionally, the lower energy expenditure caused by decreased aggression, reduced T metabolism to 11-KT, and the consequent increase in T levels during TS seems to be associated with animal growth.

Certainly, some studies have demonstrated the anabolic role of androgens in vertebrate growth^53,54^. In fish, androgen treatment increases growth^55,56^ and stimulates the Growth Hormone–Insulin-Like Growth Factor (GH-IGF) axis by activating the expression of IGF-1^57,58^. It also increases intestinal villi in Nile tilapia^59^, thereby enhancing food absorption. Thus, in the long term, increased androgens may underlie animal growth during TS.

Although no difference in length gain was observed between the treatments, significant differences were found in weight gain and SRG. This is expected because, when weight increases exponentially^60^, length increases linearly, thus demanding a longer time interval to achieve significant length differences. However, the condition factor (K) remained the same from the beginning till the end of the experimental period in the TS-treated groups but decreased in the control groups. A constant K, indicating balanced weight and length gain is usually associated with good health, production, and welfare of fish^61^. In line with this, feed efficiency was higher in the TS-treated group, even though comparable amounts of feed were offered to fish in both groups. Thus, achieving double weight gain from similar amounts of food indicates that TS is associated with better utilization of food consumed per g of fish produced Moreover, fish subjected to TS showed better survival rates, a metric that is considered an important indicator of fish welfare^62^ and productivity in aquaculture.

To conclude, long-term TS reduces aggressive interactions in Nile tilapia males. Though the treatment does not lower cortisol levels, it increases the productive performance of the fish. Growth can be considered a balancing process between energy intake and energy allocation^63^. The amounts of feed offered were similar, but food absorption is facilitated by androgen levels, thus improving energy intake. Therefore, the enhanced SGR and maintenance of K are probably a result of reduced energy allocation as overt fights were lowered. Therefore, we conclude that long-term TS provided by a simple device can be used as a tool to improve fish welfare and productivity.

## Methods

### Fish housing

Males of the genetically improved farmed tilapia (GIFT) fish, *Oreochromis niloticus* (L.), acquired from a commercial supplier, were acclimated for 20 days in indoor polypropylene tanks (approximately 500 L, 1 fish/10 L) with water at 27 °C and a photoperiod of 12 h (7:00 a.m. to 7:00 p.m.). The fish were fed twice a day (9:00 a.m. and 3:00 p.m.) with commercial feed (AGROMIX®, 32% crude protein, to apparent satiety). The water quality was maintained using biological filters (400 L/h) with constant aeration. Tanks were siphoned weekly to remove leftover food and feces, with no more than 25% of the water removed so that there was minimal harm to the chemical and social signals of the animals^33,35^.

### Experimental design

We evaluated the effects of long-term TS on aggressive interactions, social stress, and productive performance in groups of four male Nile tilapia. For this, 108 animals were assigned to any of two treatments for 21 days each: with TS (n = 13 groups; 52 individuals) and without TS (as a control, n = 14 groups; 56 animals). Mortality of animals was observed in some tanks (one dead fish per group), probably because of fights. Therefore, groups with dead participants were excluded from the analyses to avoid bias due to the number of animals and social interactions in a group. Seven groups were analyzed as the control and ten as TS-treated.

### Tactile stimulation and protocol

The fish were subjected to TS by an apparatus made of a rectangular polyvinyl chloride frame fitted with vertical plastic sticks with soft silicone bristles in their sides (Fig. 1a—apparatus used in the treatment with TS). A similar apparatus without bristles (Fig. 1b—apparatus used in the treatment without TS) was used as the control. The apparatus was introduced into the middle of each experimental aquarium (Fig. 1c) as per treatment. The fish body received TS from the bristles when it crossed the center of the aquarium (Fig. 1d). This apparatus was previously tested to ensure that neither the mucus lining of the subjects was removed, nor any injury caused to their bodies^12,26^. Furthermore, Nile tilapia spontaneously chose to cross through this type of apparatus and overcomes an aversive bright-light rout, showing motivation to access it^12^, thus ensuring its suitability for testing further hypothesis regarding TS in fish.

The experiment lasted for 21 days. Figure 1e summarizes the protocol timeline. This long-term period was chosen because it was thrice longer than the one previously used by Bolognesi et al.^26^ for Nile tilapia. On the morning of the first day, the fish were anesthetized with benzocaine (Ethyl 4-aminobenzoate, Sigma Aldrich®), at 0.03 g/L, measured, weighed, marked with colored elastomer, grouped by similar size, and assigned to one of the treatments, whose aquaria was already prepared with the respective apparatus. The animals were then video-recorded (10 min) every 3 days and on the last day (i.e., on the 1^st^, 4^th^, 7^th^, 10^th^, 13^th^, 16^th^, 19^th^, and 21^st^ days), to quantify the aggressive interactions and number of crossings through the apparatus. Videos were recorded between 2:00 p.m. and 3:00 p.m. The animals were transferred to collect blood samples (vide hormonal assay subsection below) and returned to the biometry unit on days 7 and 21 to adjust the proportion of feed offered (4% of biomass/day, divided into two meals per day, 9:00 a.m. and 3:00 p.m.) and to evaluate the productive performance of the animals, respectively. Finally, the fish were euthanized by deep anesthesia (benzocaine, 0.18 g/L) according to National Council for the Control of Animal Experimentation (CONCEA/Brazil) guidelines, as wella as ARRIVE recomendations.

Fish were individually marked with a visible implant elastomer applied under three scales at each side of the body. The animals were grouped in a 60 × 60 × 40 cm glass aquarium: approximately 140 L (1 fish/35 L). Three walls of each experimental aquarium were covered with opaque blue plastic to avoid visual contact with neighboring areas; only the front part was open for observations and filming. Blue color was used because it prevents stress in Nile tilapia^64^.

The photoperiod was set for 12 h (7:00 a.m. to 7:00 p.m.). Water parameters were monitored on day 1, 7, and 21. The mean ± S.E. of the parameter values in control and TS groups were: temperature: 27.27 ± 0.17 °C and 27.39 ± 0.16 °C; pH: 7.0 ± 0.04 in both treatments (measured by electronic device—Hanna HI98127); dissolved oxygen: 8.86 ± 0.10 ppm and 8.58 ± 0.08 ppm (measured by electronic device—Hanna HI9146); ammonia level: 0.17 ± 0.04 ppm and 0.11 ± 0.03 ppm; nitrite level:0.12 ± 0.04 ppm and 0.11 ± 0.02 ppm (measured by commercial kits—LabconTest), respectively. The animals were fed 4% of the group biomass at the same time and with the same ration provided during the “fish housing” period. The amount of feed offered during the whole experiment was similar between treatments (mean ± S.E. (g)—control: 63.95 ± 2.67 g; and TS: 54.01 ± 4.30 g; independent *t*-test, t_(15)_ = 1.76, *p* = 0.10).

### Aggressive interaction

Aggressive interactions were quantified based on the ethogram described for Nile tilapia by Carvalho & Gonçalves-de-Freitas^65^ and rated as overt or restrained fights. Overt fights are those with direct physical contact and are usually accompanied by high-energy expenditure^36^; e.g., lateral fight, mouth fight, undulation, nipping, and chase. Restrained fights, on the other hand, are aggressive displays that do not involve physical contact and are usually accompanied by low-energy expenditure ^36^; e.g., lateral threat, perpendicular threat, and circling.

### Hormonal assay

The stress level was inferred from plasma cortisol concentration^62,66^. Levels of T and its metabolite 11-KT were evaluated because of their association with aggressive behavior in fish^31^.

The animals were anesthetized by immersion in benzocaine solution (0.09 g/L), and blood was collected from the caudal vein using hypodermic needles and heparinized syringes on 7^th^ and 21^st^ days to compare their short- and long-term effects. Handling was done in less than 2 min to avoid interference with hormone levels^7^. Blood was centrifuged at 3000 rpm for 10 min, and plasma frozen at − 20 °C till further analysis. Hormone assays were performed by enzyme-linked immunosorbent assay (ELISA) using commercial kits (Cortisol: IBL Immunobiological Laboratories, Hamburg, Germany. T and 11-KT: Cayman Chemical, Michigan, USA). The hormones were analyzed in duplicate. Samples were undiluted for the cortisol assay, and 1:4 dilution was used for the T and 11-KT assays. The intra-assay and inter-assay coefficients of variation were as follows: cortisol, 6.3% and 11.2%; T, 9.6% and 9.5%; and 11-KT, 7.3% and 11.7%, respectively.

### Productive performance indicators

Productive indicators length and weight gain, SGR, and feed efficiency were used to measure the balance between the amount of food intake, metabolic rate, and energy expenditure with individual activities^67^. It was assumed that the higher the increase in these parameters, the higher will be the productive performance of fish^68^. Furthermore, condition factor (K) was calculated under the assumption that fish grow uniformly according to their length and weight^69^. These variables were calculated as follows:

$${\text{Length}}\;{\text{ gain }} = {\text{ final}}\;{\text{ standard }}\;{\text{length }}{-}{\text{ initial }}\;{\text{standard}}\;{\text{ length}}$$

$${\text{Weight}}\;{\text{gain }} = {\text{ final}}\;{\text{weight }}{-}{\text{ initial }}\;{\text{weight}}$$

$${\text{SGR }} = \, \left( {{\text{final }}\;{\text{weight }}{-}{\text{ initial}}\;{\text{ weight}}/{\text{time}}} \right).{1}00$$

Feed efficiency ratio (FER) is obtained as a ratio of weight gain/feed intake. However, we were not able to evaluate individual feed intake in the group. On the other hand, we observed that fish took the whole amount of food that was offered. Therefore, we calculate the FER as the ratio between the group’s weight gain/amount of food offered to the group.

$${\text{K }} = \, \left( {{\text{weight}}/{\text{length}}^{{3}} } \right).{1}00$$

### Statistical analysis

Data were analyzed for normality using the Kolmogorov–Smirnov test and homoscedasticity using Fmax^70^. When necessary, the data were transformed by √(x + 0.5) to adjust for homoscedasticity^71^. Mixed model ANOVA was used to compare treatments (with or without TS) and within treatments (days of observation). We analyzed the number of fish crossings using the apparatus, the number of overt and restrained fights, the level of hormones (cortisol, T, 11-KT), and initial and final standard lengths and weights. Tukey’s test for unequal samples was used as a post-hoc test. Where necessary, planned comparisons were used to compare variables of interest. An independent *t*-test was used to compare length gain, weight gain, SGR, and FER. Goodman's binomial test was performed to compare the proportion of mortality among the treatments. Statistical significance was set at *p* ≤ 0.05.

### Ethical statement

This study followed the Brazilian legislation and regulations of the National Council for the Control of Animal Experimentation (CONCEA/Brazil), being approved by the Committee on Ethics in Animal Use, IBILCE, UNESP, São José do Rio Preto, permit #213/2019. These ethical procedures are also following the ARRIVE’s guidelines, such as fish allocation in aquaria with enough space, with small gravel substrate, walls covered with blue color plastic adhesive to prevent stress, specific feeding for cichlids and maintenance of water quality. Handling for biometry and blood collection was always gentle and preceded by anesthesia in adequate concentration. At the end of the experiment, they were euthanized by deepening anesthesia, according to the CONCEA guidelines and ARRIVE recomendations. The number of replicates was adequate to answer our questions. Altogether, we avoid suffering and pain while valuing the life of fish, as well as the quality of our study.

## Data Availability

The data presented in this study are available on request from the corresponding author.
